# Identification of the miRNA–mRNA regulatory network in a mouse model of early fracture

**DOI:** 10.3389/fgene.2024.1408404

**Published:** 2024-06-11

**Authors:** Maochun Wang, Zhiyang Xie, Kaili Yan, Chongxu Qiao, Shunchao Yan, Guoping Wu

**Affiliations:** Department of Plastic Surgery, The Affiliated Friendship Plastic Surgery Hospital of Nanjing Medical University, Nanjing, China

**Keywords:** miRNA–mRNA, regulatory network, early fracture healing, transcriptome, differentially expressed genes

## Abstract

Fracture healing is a complex process that involves multiple molecular events, and the regulation mechanism is not fully understood. We acquired miRNA and mRNA transcriptomes of mouse fractures from the Gene Expression Omnibus database (GSE76197 and GSE192542) and integrated the miRNAs and genes that were differentially expressed in the control and fracture groups to construct regulatory networks. There were 130 differentially expressed miRNAs and 4,819 differentially expressed genes, including 72 upregulated and 58 downregulated miRNAs, along with 2,855 upregulated and 1964 downregulated genes during early fracture healing. Gene ontology analysis revealed that most of the differentially expressed genes were enriched in the extracellular matrix (ECM) structure and the ECM organization. The Kyoto Encyclopedia of Genes and Genomes (KEGG) enrichment suggested cell cycle, DNA replication, and mismatch repair were involved in the progression of fracture healing. Furthermore, we constructed a molecular network of miRNAs and mRNAs with inverse expression patterns to elucidate the molecular basis of miRNA–mRNA regulation in fractures. The regulatory network highlighted the potential targets, which may help to provide a mechanistic basis for therapies to improve fracture patient outcomes.

## Introduction

Bone fractures are one of the most common injuries in humans; approximately 3.21 of every 1,000 people are affected by fractures every year (Chen et al., 2017). Fracture is the main cause of death and disability worldwide, bringing a huge economic burden to society and families (Wu et al., 2021). Many risk factors affect fracture healing, including age, infection, vascular distribution, immune response, and fixation of the fracture (Cheng and Shoback, 2019; Zhang et al., 2021). While most bone fractures heal normally, approximately 1.9%–4.9% result in non-union (Wildemann et al., 2021), and the non-union patients undergo more long-term pain and mental health issues (Zura et al., 2016).

Fracture healing generally comprises four stages: hematoma formation, granulation tissue formation, callus formation, and bone remodeling (Claes et al., 2012; Einhorn and Gerstenfeld, 2015). It involves a series of complex physiological and pathological events, multiple tissues and cells, and many signaling pathways, like BMP, WNT, and inflammatory signaling pathways (Deschaseaux et al., 2009). However, the specific molecular mechanisms involved in fracture healing are not yet clear.

MicroRNAs (miRNAs) are a class of short non-coding RNA (approximately 18–25 nucleotides) that regulate gene expression by binding the 3′-untranslated region of the target mRNAs, resulting in translational inhibition or target degradation (Jing et al., 2015; Plotkin and Wallace, 2021). It has been shown that miRNA plays key roles in fracture healing, osteogenesis, and bone homeostasis (Lian et al., 2012; Nugent, 2017). Injection of miR-29b-3p in mice improves femoral fracture healing by promoting osteogenesis of bone marrow-derived mesenchymal stem cells (BMSCs) (Lee et al., 2016). miR-223-3p shows high expression in fracture patients, and it regulates osteoblast activity and apoptosis by targeting FGFR2 (Wang et al., 2021). Plasma miR-92a plays an important role in human fracture healing, and its inhibition promotes fracture healing through angiogenesis (Murata et al., 2014). It has also been shown that the miRNA–mRNA network has a regulatory role in osteoblast differentiation, the pathogenesis of osteoporosis, and fracture healing (An et al., 2014; Zhang et al., 2020; Li et al., 2021; Mohanapriya et al., 2022). Based on the advantages of miRNA, transcriptome analysis combining miRNA and mRNA of fractures will help to identify potential therapeutic targets for fracture healing.

Here, we integrated miRNA and mRNA transcription profiles of early fractures in mice to construct regulatory networks and identified potential molecular crosstalk during fracture healing, which could provide insights for future fracture treatment.

## Material and methods

### Transcriptome quality control

Transcriptomes of miRNA and mRNA in early fractures were acquired from the Gene Expression Omnibus (GEO) database, including mRNA (GSE192542, Control = 6, Fracture = 6) (Shainer et al., 2023) and miRNA (GSE76197, Control = 3, Fracture = 3) (Hadjiargyrou et al., 2016). The selection of these data was based on the following principles, including data sourced from the broken femurs of C57BL/6 mice. The miRNA and mRNA data were the transcriptome at 3 days after fracture. We chose the above two datasets to ensure consistency and accuracy in data analysis. According to the description of the datasets, the control and fracture samples were collected from the right femur bone of C57BL/6 male mice with the age of 6–7 weeks. A medial patellar incision was made, followed by patellar dislocation and exposure of the femoral condyle. Muscles and tendons were removed to expose the bone shaft, and a horizontal fracture was created at the center with surgical scissors and fixed with a needle (Shainer et al., 2023). All samples were collected 3 days after surgery, and RNA was extracted to obtain transcriptome data.

Raw counts of mRNA transcriptome from Illumina NextSeq 500 were analyzed with the DESeq2 (v1.40.2) R package, with counts converted to TPM for data quality assessment, obtaining 17,687 unique genes. Raw data of the miRNA transcriptome from Agilent-070155 Mouse miRNA Microarray was read and normalized by the AgiMicroRna (v2.50.0) R package. Probes were transformed into gene symbols in which expression values were calculated by the average method, resulting in 729 unique miRNAs for further analysis. Principal component analysis (PCA) of miRNA and mRNA was performed using the factoextra (v1.0.7) and FactoMineR (v2.9) R packages.

### Identification and analysis of differentially expressed miRNAs and mRNAs

Differentially expressed miRNAs and mRNAs with a twofold expression difference and a *p*-value less than 0.05 were screened by the same criteria. A volcano plot was illustrated using ggplot2 (v3.4.4), and the top 10 differentially expressed genes (DEGs) were labeled. A heatmap of the top 10 DEGs was created using pheatmap (v1.0.12). All DEGs were further enriched by the biological process of Gene Ontology (GO), Kyoto Encyclopedia of Genes and Genomes (KEGG), and Gene Set Enrichment Analysis (GSEA) with the clusterProfiler (v4.8.3) and enrichplot (v1.20.3) R packages. The results of GO and KEGG were analyzed based on the criteria of pvalueCutoff = 0.05 and qvalueCutoff = 0.2, and the top 10 biological processes of GO and the top 15 pathways of KEGG were presented.

### qRT-PCR validation in mRNA transcriptome

All animal procedures were performed in the animal experiment center of the Affiliated Friendship Plastic Surgery Hospital of Nanjing Medical University. Six-week-old C57BL/6 male mice received femur bone fracture surgery, in which a horizontal fracture was created at the center with surgical scissors and fixed with a needle. A medial patellar incision was made in the control mice to expose the femoral bone shaft. The incision was immediately stitched without any further operation. The right femur bone shaft was harvested 3 days post-surgery. RNA was extracted with Trizol (Thermo Fisher Scientific), and 1 μg RNA was reverse-transcribed by a RevertAid First Strand cDNA Synthesis Kit (Thermo Fisher Scientific). The expression of four genes, including Cdh5, Reln, Mapt, and Atg9a, were verified by qRT-PCR. The primers were: Cdh5 (forward, 5′-CCA​CTG​CTT​TGG​GAG​CCT​T-3’; reverse, 5′-GGC​AGG​TAG​CAT​GTT​GGG​G-3′), Reln (forward, 5′- TTA​CTC​GCA​CCT​TGC​TGA​AAT-3’; reverse, 5′- CAG​TTG​CTG​GTA​GGA​GTC​AAA​G-3′), Mapt (forward, 5′- ACC​CCA​TCC​CTA​CCA​ACA​C -3’; reverse, 5′- CAG​GCG​GCT​CTT​ACT​AGC​TG-3′) and Atg9a (forward, 5′- ATG​GCT​CTC​TTA​TCA​CCA​TCC​T -3’; reverse, 5′- TGG​ATC​TCC​CAA​TAG​CAG​CAA-3′). cDNA was diluted 10 times, and four replications of qRT-PCR were performed with ChamQ SYBR Color qPCR Master Mix (Vazyme) on an ABI 7500 (Applied Biosystems). Relative gene expression was calculated using the 2^−ΔΔCt^ method, and β-actin was used as the internal control. Relative expression data were shown as mean ± SD by GraphPad Prism 9.

### Target prediction of miRNAs with TargetScan and miRDB

miRNA targets were predicted using default parameters in the TargetScan (Release 7.1) and miRDB (https://mirdb.org/) databases. VennDiagram (v1.7.3) software was used to intersect the targets and DEGs obtained from TargetScan and miRDB to acquire common genes with altered expression in fractures.

### Construction of the miRNA–mRNA network

miRNAmRNA pairs with shared targets in TargetScan, miRDB, and DEGs were filtered based on miRNA’s negative regulatory mechanism and the opposite expression trends of miRNA and mRNA. Filtered miRNA–mRNA pairs were imported into Cytoscape (v3.9.0) to construct an miRNA–mRNA regulatory network. miRNAs were marked with cyan, upregulated genes were marked with light red, and downregulated genes were marked with light purple. Expression values of hub miRNAs and mRNAs were shown as mean ± SD, and statistical analysis was made by unpaired t-tests on GraphPad Prism 9 (v9.3.1). ns represented no statistical significance. * represented statistical significance.

## Results

### Screening of differentially expressed genes in early fracture healing in mice

**FIGURE 1:**
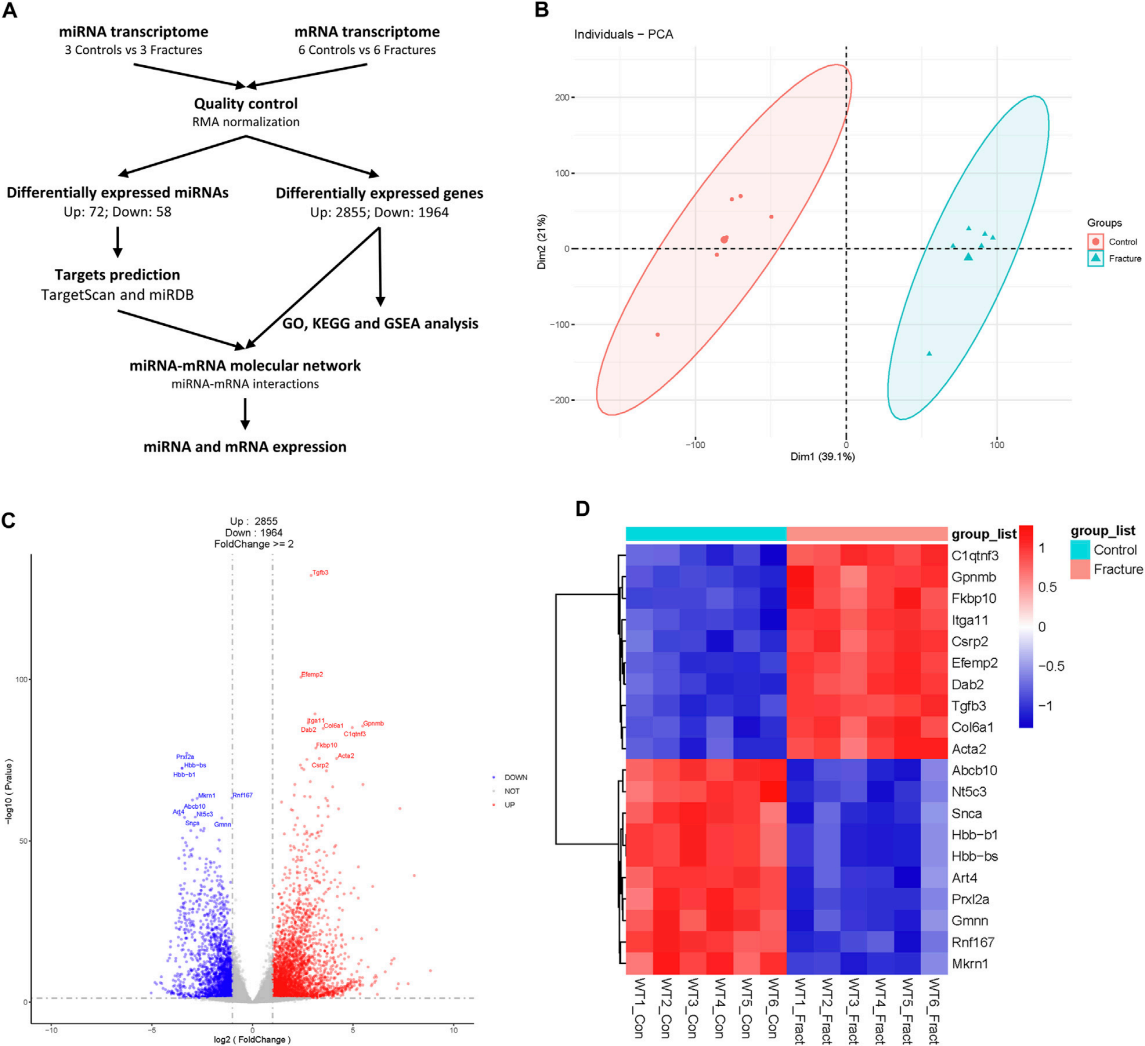
Process diagram and analysis of mouse fracture mRNA transcriptome. **(A)** A schematic of this study. **(B)** Principal component analysis of mouse control and fracture groups’ mRNA transcriptomes. Circles represent the control group, and triangles represent the fracture group. **(C)** Volcano plot of mRNA transcriptome. Red indicates upregulated genes, and blue indicates downregulated genes. The top 10 differentially expressed genes are labeled. **(D)** Heatmap of top 10 DEGs with statistical differences.

In general, we used the miRNA and mRNA transcriptome to identify differentially expressed miRNAs and mRNAs and constructed an miRNA–mRNA regulatory network to reveal transcriptional mechanisms during early fracture healing in mice (Figure 1A).

The boxplot showed the signal levels were uniform after normalization (Supplementary Figure S1A). PCA analysis showed that control and fracture data were clustered separately, which indicated the transcriptome similarity of the control and fracture groups and the difference between the control and fracture groups (Figure 1B). We also confirmed with a cluster dendrogram (Supplementary Figure S1B). A total of 4,819 DEGs were identified, with 2,855 upregulated and 1964 downregulated in fractures compared to controls (Figure 1C; Supplementary Table S1). Gpnmb, Itga11, Tgfb3, and Col6a1 were upregulated, and Abcb10, Art4, Prxl2a, and Rnf167 were downregulated during early fracture healing in mice (Figure 1D). Tgfb3 and Itga11 were significantly upregulated in fractures (Figures 1C,D), highlighting the crucial role of TGFβ and MAPK signaling pathways in early fracture healing (Poniatowski et al., 2015; Chow et al., 2021).

### Gene enrichment of mRNA transcriptome

**FIGURE 2:**
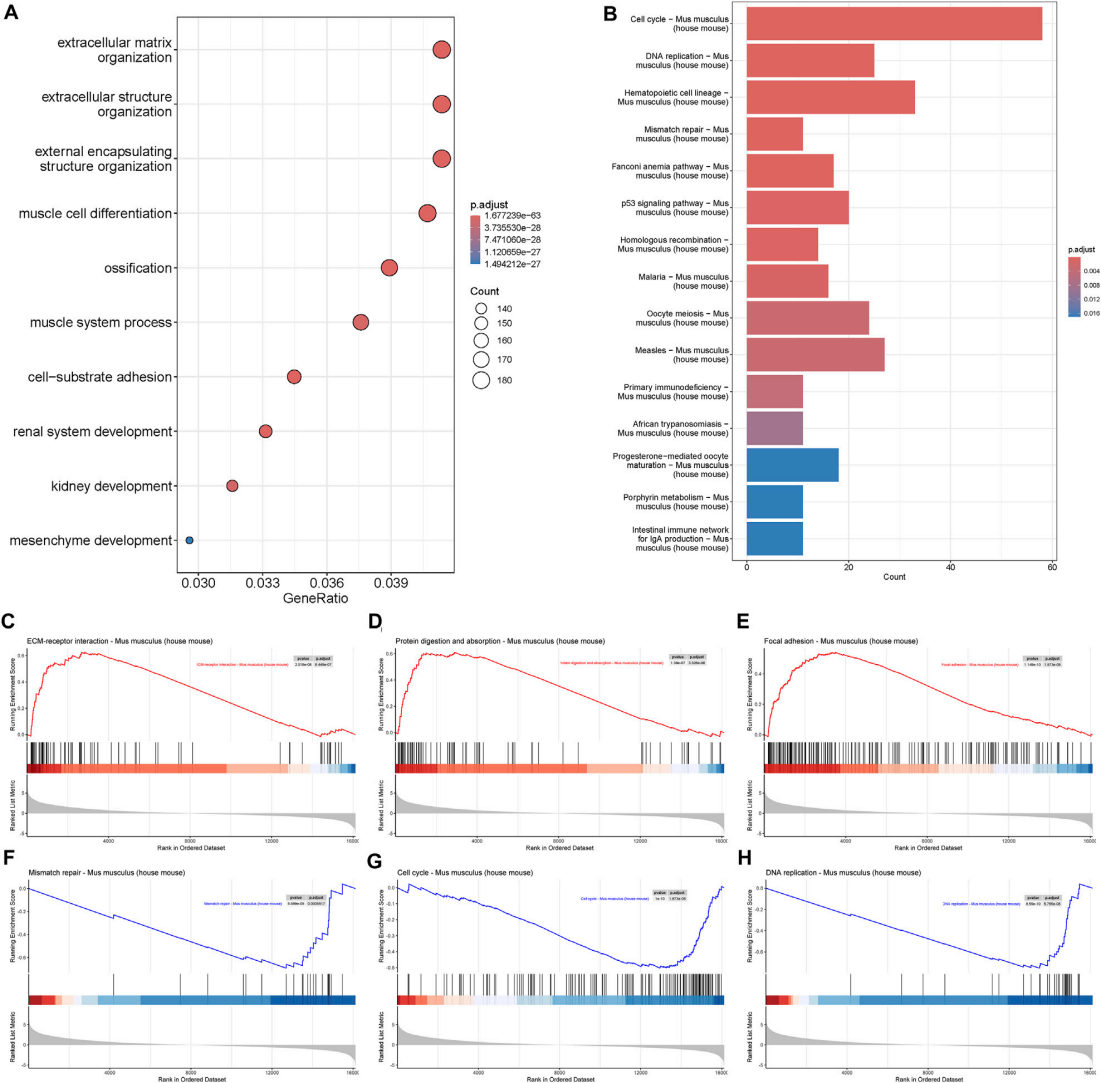
Gene enrichment of mRNA transcriptome. **(A)** Biological process of Gene Ontology (GO), and **(B)** Kyoto Encyclopedia of Genes and Genomes (KEGG) analysis of differentially expressed genes during fracture healing. **(C–E)** Gene Set Enrichment Analysis (GSEA) of upregulated genes in mouse fracture. **(F–H)** GSEA of downregulated genes in mouse fracture.

GO biological process analysis showed that DEGs were mostly enriched in extracellular matrix organization and extracellular structure organization (Figure 2A), which was consistent with the crucial role of the ECM in bone integrity and strength (Alcorta-Sevillano et al., 2020). Some genes were enriched in muscle process and mesenchyme development, which demonstrated the importance of muscle and mesenchyme cells during early fracture healing. KEGG showed that DEGs were associated with cell cycle, DNA replication, and mismatch repair, suggesting an alteration in cell proliferation activity in the early stage of fracture healing (Figure 2B). GSEA showed that upregulated genes were related to ECM-receptor interaction, protein digestion and absorption, and focal adhesion, and downregulated genes were related to mismatch repair, cell cycle, and DNA replication (Figures 2C–H). These results suggested an increase in cell communication activity and a decrease in cell proliferation activity during early fracture healing.

### Differentially expressed miRNAs and target prediction

**FIGURE 3:**
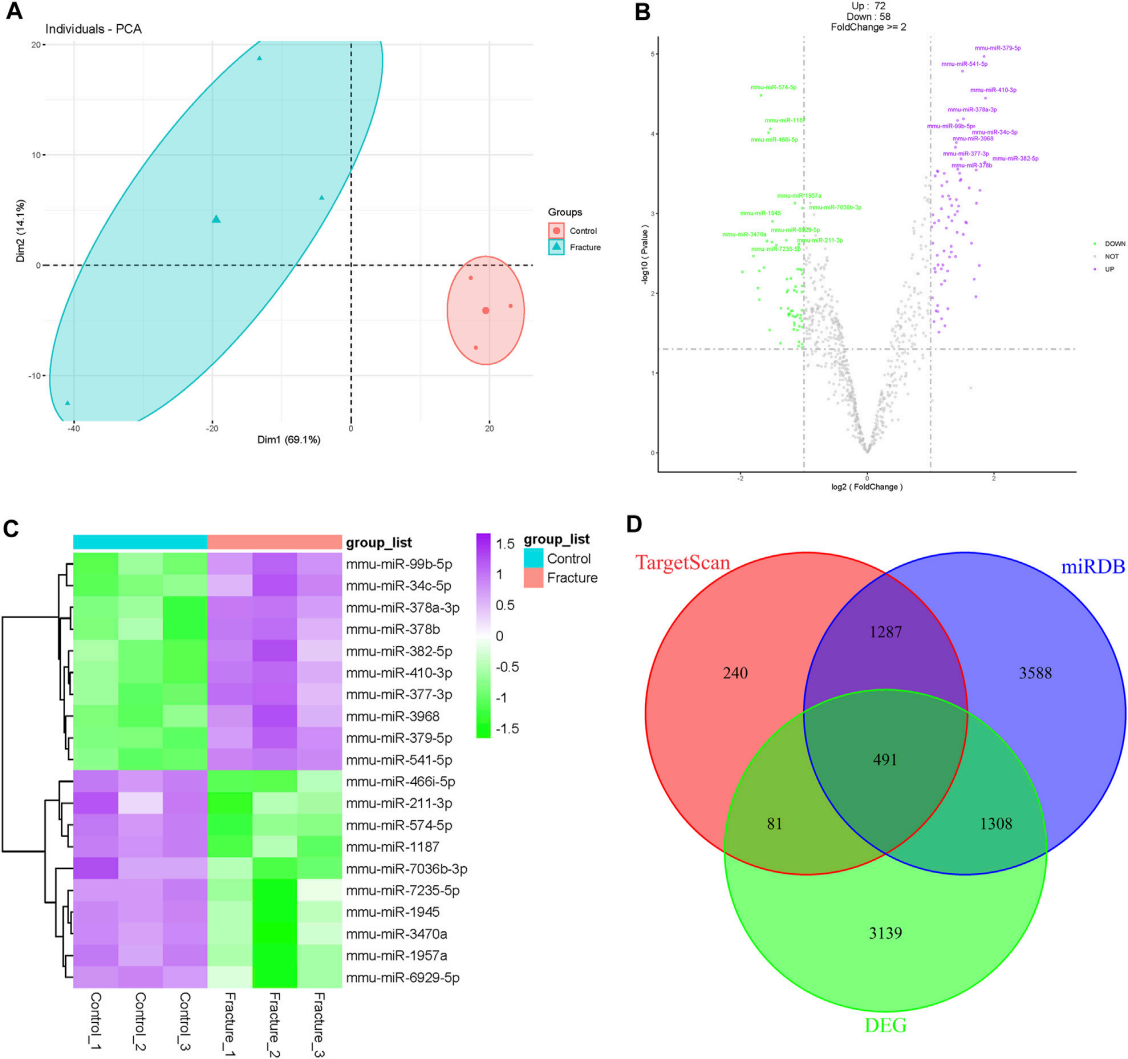
Analysis of mouse fracture miRNA transcriptome. **(A)** Principal component analysis of mouse control and fracture groups’ miRNA transcriptomes. The circles represent the control group, and the triangles represent the fracture group. **(B)** Volcano plot of miRNA transcriptome. Purple indicates upregulated miRNAs, and green indicates downregulated miRNAs. The top 10 differentially expressed miRNAs are labeled. **(C)** Heatmap of top 10 differentially expressed miRNAs with statistical differences. **(D)** Venn diagram of differentially expressed genes and targets predicted in TargetScan and miRDB.

Similar to the mRNA transcriptome, probe signals of the miRNA transcriptome were uniform after RMA normalization (Supplementary Figure S1C), and the control and fracture groups were clustered separately in PCA and the cluster dendrogram (Figure 3A; Supplementary Figure S1D). Although there were some differences within the fracture group, they were separated from the control group overall. There were 130 differentially expressed miRNAs, including 72 upregulated miRNAs and 58 downregulated miRNAs during early fracture healing (Figure 3B; Supplementary Table S2). miR-378a-3p, miR-410-3p, miR-379-5p, and miR-541-5p were upregulated, and miR-466i-5p, miR-574-5p, miR-1187 and miR-1957a were downregulated during early fracture healing in mice (Figure 3C). Targets of all differentially expressed miRNAs were predicted in the TargetScan and miRDB databases. There were 2099 candidates in TargetScan and 6,674 candidates in miRDB, which overlapped 491 DEG candidates (Figure 3D).

### miRNA–mRNA molecular network

**FIGURE 4:**
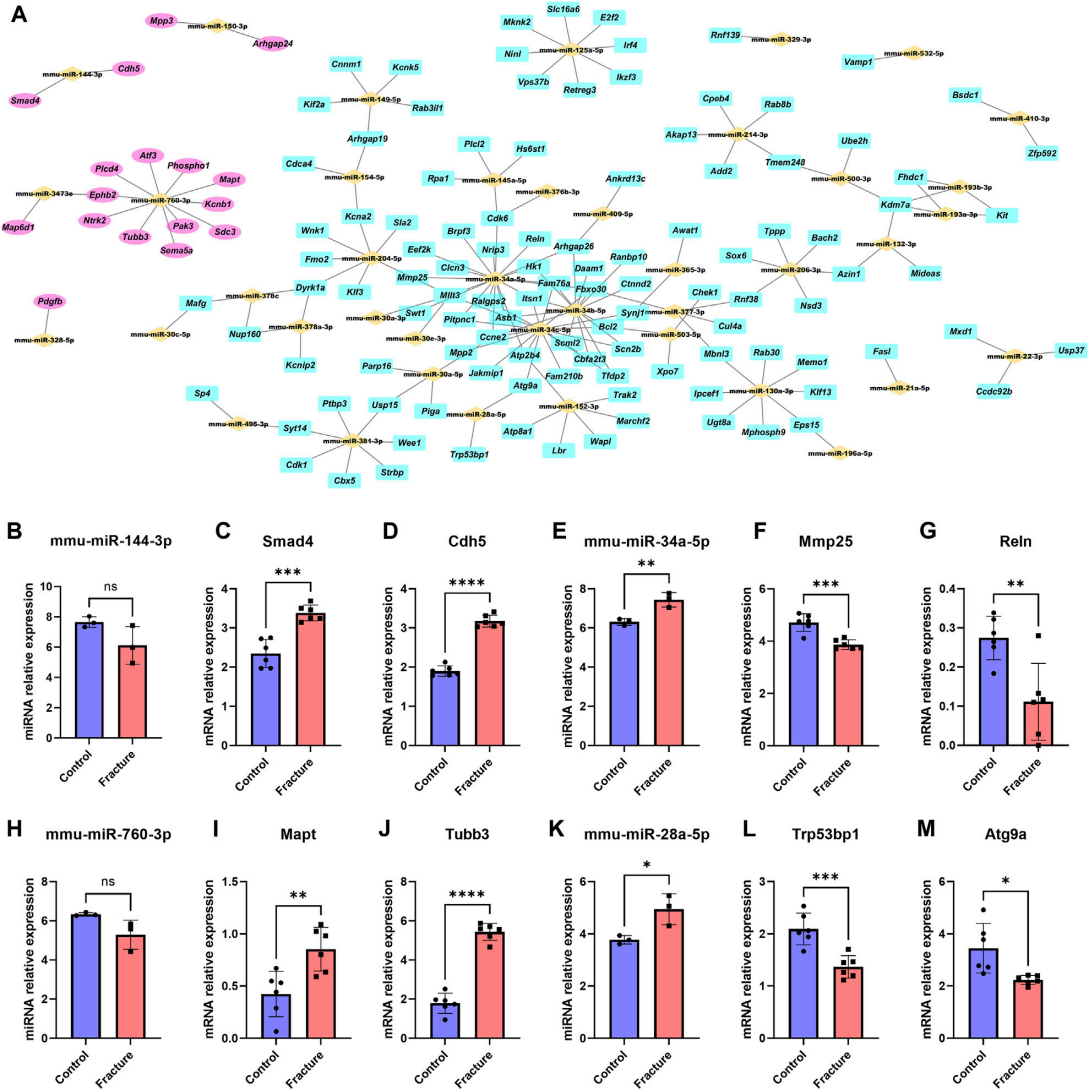
miRNA–mRNA molecular network. **(A)** Potential molecular crosstalk of miRNA and mRNA during mouse fracture healing. miRNAs are represented by yellow bricks, upregulated genes are represented by pink ellipses, and downregulated genes are represented by cyan rectangles. The expression trends of miRNA and mRNA move in opposite directions after fracture. **(B–M)** Relative expression of miR-144-3p **(B)**, Smad4 **(C)**, Cdh5 **(D)**, miR-34a-5p **(E)**, Mmp25 **(F)**, Reln **(G)**, miR-760-3p **(H)**, Mapt **(I)**, Tubb3 **(J)**, miR-28a-5p **(K)**, Trp53bp1 **(L)**, and Atg9a **(M)** between the control and fracture groups. Data are presented as the mean ± SD. * represents statistical significance. Yellow represents the control group, and red represents the fracture group.

Based on the negative miRNA regulation mechanism, 491 miRNA–mRNA pairs were further screened to 179 pairs with opposite expressions of miRNA and mRNA. We imported these paired and differentially expressed miRNAs and mRNA into Cytoscape software to build an miRNA–mRNA regulatory network (Figure 4A). miR-760-3p, miR-144-3p, and miR-150-3p played a central role in upregulated genes, while miR-34a-5p, miR-34b-5p, miR-34c-5p, and miR-28a-5p were at the center of downregulated genes. We also found that the expression of miR144-3p and miRNA-760-3p decreased in fracture, while osteogenesis-associated Smad4 and Cdh5 and microtubule-associated Mapt and Tubb3 increased in fracture (Figures 4B–D and Figures 4H–J). Expression of miR34a-5p and miRNA-28a-5p increased, while expression of catabolism-associated Mmp25 and Reln and cell proliferation-associated Trp54bp1 and Atg9a decreased in fracture (Figures 4E–G and Figure 4K–M). We further selected four genes for qPCR validation in the mouse fracture model. The expression of Cdh5 and Mapt was increased, while the expression of Reln and Atg5a decreased, which was consistent with the findings of our mRNA transcriptome analysis (Supplementary Figure S1E–H).

## Discussion

The healing of bone fractures depends on a series of complex events. Hematoma and granulation occur in the early stage of fracture, and inflammation can mediate and activate the proliferation of bone progenitor/stem cells, which further differentiate into chondrocytes and osteoblasts and form a callus at the fracture site (Shainer et al., 2023). As an important regulatory mechanism of gene transcription, miRNAs play crucial roles in osteogenesis (Dong et al., 2012; Moghaddam and Neshati, 2019). Here, we integrated the miRNA and mRNA transcriptome of fractures and constructed an miRNA–mRNA regulatory network for early fracture in a mouse model.

All transcriptome data showed basic reliability after quality control. The box plots of miRNA and mRNA transcriptomes showed that the signal values were uniform after standardization, and the median of the data was basically consistent. Further PCA analysis showed intragroup clustering and intergroup separation between the control and fracture groups, indicating transcriptome similarity of the control and fracture groups and the difference between the control and fracture groups. The results of the cluster dendrogram also indicated this point. However, in the PCA analysis of miRNA transcriptome, although the fracture group was separated from the control group, the clustering degree within the fracture group was slightly poor, which might be influenced by the sampling process after fracture or the complexity of fracture repair itself.

The ECM was crucial for bone integrity and strength. We found that the DEGs in mRNA transcriptome analysis of fractures were mainly enriched in extracellular matrix organization and extracellular structure organization biological processes. At the same time, the enriched biological processes of muscle cell differentiation, muscle system process, and mesenchyme development suggested that muscle and mesenchyme cells were involved in the early fracture healing process. Interestingly, GSEA analysis showed that most genes in the ECM-receptor interaction, protein digestion and absorption, and focal adhesion were upregulated, indicating communication between cells and the external environment, communication between different cells, and cell homeostasis. Most genes in mismatch repair, cell cycle, and DNA replication were downregulated, indicating decreased cell proliferation ability, which contradicted our general knowledge. This might be due to proliferation reaching the peak in mice 3 days after fracture, or the gait and load dynamics of non-fractured mice might alter gene expression (Hadjiargyrou et al., 2016).

Currently, research on the regulatory network of miRNA–mRNA in fracture healing is limited, especially in the early stage of fracture. Our results systematically demonstrated the changes in the miRNA and mRNA transcriptome and regulatory network in the early stage of fracture healing in mice, which supplemented the mechanism research of miRNA regulation in the fracture healing field. We found that miR-144-3p was downregulated in the early stage of fracture, while its candidate target gene, Smad4, was upregulated. Research has shown that miR-144-3p inhibits bone formation during distraction osteogenesis (Sun et al., 2017), while Smad4 expression is upregulated in rat fracture callus (Yu et al., 2002), and the Smad4 signaling pathway can promote tibial fracture healing in mice (Wang et al., 2022). These results were consistent with our observations. Other studies found that miR-34a-5p facilitates osteogenic differentiation of bone marrow mesenchymal stem cells and modulates bone metabolism in mice (Sun et al., 2023). miR-34c-5p can promote osteogenic differentiation of bone marrow mesenchymal stem cells in rabbits (Liu et al., 2021), which was in line with our findings that miR-34a-5p and miR-34c-5p were upregulated after fracture and play an important role in the process of fracture healing. Meanwhile, we found that miR-760-3p was downregulated in the early stage of fracture healing, and its candidate target genes, Mapt and Tubb3, were upregulated. Mapt and Tubb3 were microtubule-related proteins, and there was no relevant research showing the relationship between microtubule homeostasis and fracture healing. Our findings suggested that miR-760-3p might interfere with the fracture healing process by targeting Mapt and Tubb3 to affect microtubule homeostasis, which provided new directions for fracture research.

However, this study still had some limitations. First, the small miRNA transcriptome sample size might result in many potentially differential miRNAs being excluded from the screening criteria. Second, the transcriptome of mRNA came from RNA-seq, while the transcriptome of miRNA came from a microarray. RNA-seq could detect almost all mRNA with poly(A) tails, but the sequencing process was influenced by PCR preference. The microarray detection had strong stability but was limited by the number of probes. Finally, all miRNA–mRNA networks were analyzed by bioinformatics, but they might not necessarily be the exact targets of every miRNA. Additional research is necessary to validate the predicted target genes through experiments on the dual luciferase reporting system. Our future research aims to validate these functions.

We analyzed the differential expression of miRNA and mRNA in early fracture healing in a mouse model and integrated the transcriptome of miRNA and mRNA to construct a regulatory network of miRNA–mRNA. We identified potential molecular crosstalk during fracture healing, which provides insights for future fracture treatment.

## Data Availability

The data presented in the study are deposited in Gene Expression Omnibus, and the access numbers are GSE76197 and GSE192542.

## References

[B1] Alcorta-Sevillano N. Macías I. Infante A. Rodríguez C. I. (2020). Deciphering the relevance of bone ECM signaling. Cells 9 (12), 2630. 10.3390/cells9122630 33297501 PMC7762413

[B2] An J. H. Ohn J. H. Song J. A. Yang J.-Y. Park H. Choi H. J. (2014). Changes of MicroRNA profile and MicroRNA-mRNA regulatory network in bones of ovariectomized mice. J. Bone Mineral Res. 29 (3), 644–656. 10.1002/jbmr.2060 23929739

[B3] Chen W. Lv H. Liu S. Liu B. Zhu Y. Chen X. (2017). National incidence of traumatic fractures in China: a retrospective survey of 512 187 individuals. Lancet Glob. Health 5 (8), e807–e817. 10.1016/S2214-109X(17)30222-X 28666814

[B4] Cheng C. Shoback D. (2019). Mechanisms underlying normal fracture healing and risk factors for delayed healing. Curr. Osteoporos. Rep. 17, 36–47. 10.1007/s11914-019-00501-5 30671884

[B5] Chow S. K. H. Cui C. Cheng K. Y. K. Chim Y. N. Wang J. Wong C. H. W. (2021). Acute inflammatory response in osteoporotic fracture healing augmented with mechanical stimulation is regulated *in vivo* through the p38-MAPK pathway. Int. J. Mol. Sci. 22 (16), 8720. 10.3390/ijms22168720 34445423 PMC8395718

[B6] Claes L. Recknagel S. Ignatius A. (2012). Fracture healing under healthy and inflammatory conditions. Nat. Rev. Rheumatol. 8 (3), 133–143. 10.1038/nrrheum.2012.1 22293759

[B7] Deschaseaux F. Sensébé L. Heymann D. (2009). Mechanisms of bone repair and regeneration. Trends Mol. Med. 15 (9), 417–429. 10.1016/j.molmed.2009.07.002 19740701

[B8] Dong S. Yang B. Guo H. Kang F. (2012). MicroRNAs regulate osteogenesis and chondrogenesis. Biochem. Biophysical Res. Commun. 418 (4), 587–591. 10.1016/j.bbrc.2012.01.075 22306817

[B9] Einhorn T. A. Gerstenfeld L. C. (2015). Fracture healing: mechanisms and interventions. Nat. Rev. Rheumatol. 11 (1), 45–54. 10.1038/nrrheum.2014.164 25266456 PMC4464690

[B10] Hadjiargyrou M. Zhi J. Komatsu D. E. (2016). Identification of the microRNA transcriptome during the early phases of mammalian fracture repair. Bone 87, 78–88. 10.1016/j.bone.2016.03.011 27058875

[B11] Jing D. Hao J. Shen Y. Tang G. Li M.-L. Huang S.-H. (2015). The role of microRNAs in bone remodeling. Int. J. oral Sci. 7 (3), 131–143. 10.1038/ijos.2015.22 26208037 PMC4582559

[B12] Lee W. Y. Li N. Lin S. Wang B. Lan H. Y. Li G. (2016). miRNA-29b improves bone healing in mouse fracture model. Mol. Cell. Endocrinol. 430, 97–107. 10.1016/j.mce.2016.04.014 27113026

[B13] Li X. Zhong Z. Ma E. Wu X. (2021). Identification of miRNA regulatory networks and candidate markers for fracture healing in mice. Comput. Math. Methods Med. 2021, 2866475–2866513. 10.1155/2021/2866475 34840596 PMC8611357

[B14] Lian J. B. Stein G. S. van Wijnen A. J. Stein J. L. Hassan M. Q. Gaur T. (2012). MicroRNA control of bone formation and homeostasis. Nat. Rev. Endocrinol. 8 (4), 212–227. 10.1038/nrendo.2011.234 22290358 PMC3589914

[B15] Liu B. Gan W. Jin Z. Wang M. Cui G. Zhang H. (2021). The role of miR-34c-5p in osteogenic differentiation of bone marrow mesenchymal stem cells. Int. J. Stem Cells 14 (3), 286–297. 10.15283/ijsc20188 33906980 PMC8429940

[B16] Moghaddam T. Neshati Z. (2019). Role of microRNAs in osteogenesis of stem cells. J. Cell. Biochem. 120 (8), 14136–14155. 10.1002/jcb.28689 31069839

[B17] Mohanapriya R. Akshaya R. L. Selvamurugan N. (2022). A regulatory role of circRNA-miRNA-mRNA network in osteoblast differentiation. Biochimie 193, 137–147. 10.1016/j.biochi.2021.11.001 34742858

[B18] Murata K. Ito H. Yoshitomi H. Yamamoto K. Fukuda A. Yoshikawa J. (2014). Inhibition of miR-92a enhances fracture healing via promoting angiogenesis in a model of stabilized fracture in young mice. J. Bone Mineral Res. 29 (2), 316–326. 10.1002/jbmr.2040 23857760

[B19] Nugent M. (2017). MicroRNAs and fracture healing. Calcif. tissue Int. 101 (4), 355–361. 10.1007/s00223-017-0296-x 28589206

[B20] Plotkin L. I. Wallace J. M. (2021). MicroRNAs and osteocytes. Bone 150, 115994. 10.1016/j.bone.2021.115994 33965651 PMC8217311

[B21] Poniatowski Ł. A. Wojdasiewicz P. Gasik R. Szukiewicz D. (2015). Transforming growth factor Beta family: insight into the role of growth factors in regulation of fracture healing biology and potential clinical applications. Mediat. Inflamm. 2015, 137823. 10.1155/2015/137823 PMC4325469 25709154

[B22] Shainer R. Kram V. Kilts T. M. Li L. Doyle A. D. Shainer I. (2023). Biglycan regulates bone development and regeneration. Front. Physiol. 14, 1119368. 10.3389/fphys.2023.1119368 36875017 PMC9979216

[B23] Sun D. Chen Y. Liu X. Huang G. Cheng G. Yu C. (2023). miR-34a-5p facilitates osteogenic differentiation of bone marrow mesenchymal stem cells and modulates bone metabolism by targeting HDAC1 and promoting ER-α transcription. Connect. Tissue Res. 64 (2), 126–138. 10.1080/03008207.2022.2108415 36537660

[B24] Sun Y. X. Zhang J. F. Xu J. Xu L. L. Wu T. Y. Wang B. (2017). MicroRNA-144-3p inhibits bone formation in distraction osteogenesis through targeting Connexin 43. Oncotarget 8 (52), 89913–89922. 10.18632/oncotarget.20984 29163798 PMC5685719

[B25] Wang B. Wu W. Xu K. Wu H. (2021). MicroRNA-223-3p is involved in fracture healing by regulating fibroblast growth factor receptor 2. Bioengineered 12 (2), 12040–12048. 10.1080/21655979.2021.2002498 34753389 PMC8810112

[B26] Wang J. Wei Y. Zhou Z. Yang J. Jia Y. Wu H. (2022). Deer antler extract promotes tibia fracture healing in mice by activating BMP-2/SMAD4 signaling pathway. J. Orthop. Surg. Res. 17 (1), 468. 10.1186/s13018-022-03364-2 36307889 PMC9617435

[B27] Wildemann B. Ignatius A. Leung F. Taitsman L. A. Smith R. M. Pesántez R. (2021). Non-union bone fractures. Nat. Rev. Dis. Prim. 7 (1), 57. 10.1038/s41572-021-00289-8 34354083

[B28] Wu A.-M. Bisignano C. James S. L. Abady G. G. Abedi A. Abu-Gharbieh E. (2021). Global, regional, and national burden of bone fractures in 204 countries and territories, 1990–2019: a systematic analysis from the Global Burden of Disease Study 2019. Lancet Healthy Longev. 2 (9), e580–e592. 10.1016/S2666-7568(21)00172-0 34723233 PMC8547262

[B29] Yu Y. Yang J.-L. Chapman-Sheath P. J. Walsh W. R. (2002). TGF-beta, BMPS, and their signal transducing mediators, Smads, in rat fracture healing. J. Biomed. Mater. Res. 60 (3), 392–397. 10.1002/jbm.1289 11920662

[B30] Zhang H. Wang R. Wang G. Zhang B. Wang C. Li D. (2021). Single-cell RNA sequencing reveals B cells are important regulators in fracture healing. Front. Endocrinol. 12, 666140. 10.3389/fendo.2021.666140 PMC8606664 34819916

[B31] Zhang Z. Yue L. Wang Y. Jiang Y. Xiang L. Cheng Y. (2020). A circRNA-miRNA-mRNA network plays a role in the protective effect of diosgenin on alveolar bone loss in ovariectomized rats. BMC Complementary Med. Ther. 20 (1), 220. 10.1186/s12906-020-03009-z PMC7362493 32664914

[B32] Zura R. Xiong Z. Einhorn T. Watson J. T. Ostrum R. F. Prayson M. J. (2016). Epidemiology of fracture nonunion in 18 human bones. JAMA Surg. 151 (11), e162775. 10.1001/jamasurg.2016.2775 27603155

