# Supplementary figures and images for: Identification of the miRNA–mRNA regulatory network in a mouse model of early fracture
Source: Front Genet. 2024 Jun 11;15:1408404. doi: 10.3389/fgene.2024.1408404 (PMC11196604; doi:10.3389/fgene.2024.1408404)

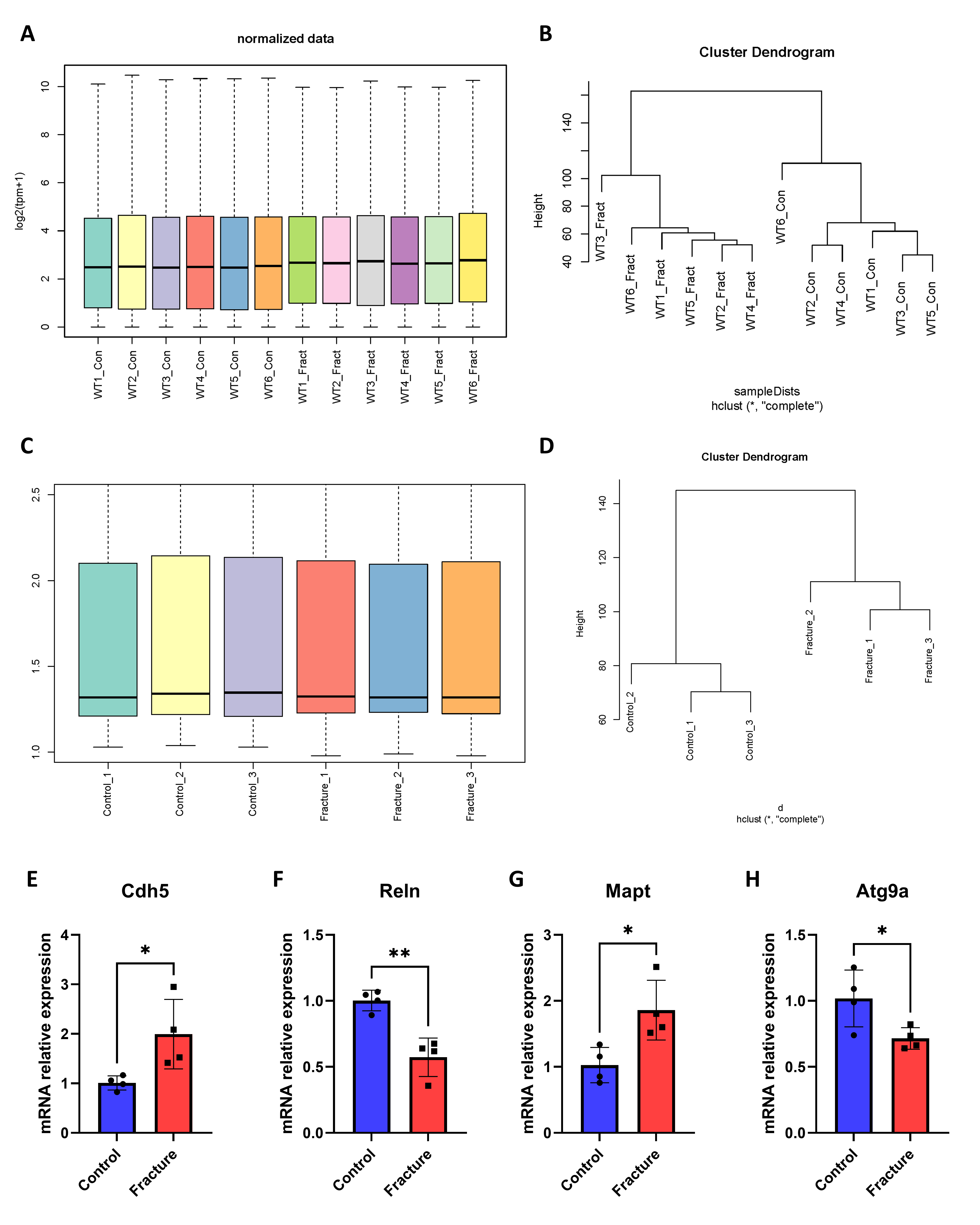

Supplement: Supplementary file 2 [file Image1.TIF]
